# Inhibition of Ornithine Decarboxylase 1 Mitigates Denervation‐Induced Muscle Atrophy by Suppressing Proteolysis and Preserving Muscle Stem Cell Homeostasis

**DOI:** 10.1111/cpr.70231

**Published:** 2026-05-15

**Authors:** Mingming Zhang, Feifan Chang, Siliang Ge, Fuming Cao, Pincong Fu, Haobo Zhang, Ming Chen, Yi Li, Peifu Tang, Pengbin Yin

**Affiliations:** ^1^ Senior Department of Orthopedics The Fourth Medical Center of Chinese PLA General Hospital Beijing China; ^2^ National Clinical Research Center for Orthopedics, Sports Medicine & Rehabilitation Beijing China; ^3^ School of Medicine Nankai University Tianjin China

**Keywords:** denervation, muscle atrophy, muscle stem cell, polyamine metabolism

## Abstract

Neural innervation is of vital importance for muscle mass and function, and denervation induces progressive muscle atrophy, which lacks effective treatments. Polyamine metabolism is reported to be innervation responsive and involved in denervation‐induced muscle atrophy, yet the effects of polyamine in denervated muscle atrophy remain unknown. In this study, using a sciatic nerve transection model, we observed progressive increases in putrescine and spermidine, alongside induction of polyamine metabolism enzymes, coincident with the early rapid atrophy phase post denervation. Pharmacological ODC1 inhibition lowered intramuscular polyamines and improved denervated muscle atrophy and fibrosis. In contrast, spermidine supplementation induced muscle atrophy and atrogene expression in healthy and denervated muscle, implicating spermidine as a pro‐atrophic metabolite. Single‐nucleus sequencing revealed an expansion of atrophic myonuclei and depletion of type IIb myonuclei post denervation. DFMO reduced the atrophic myonuclear fraction, increased MuSCs abundance, and suppressed FAPs derived FGF signalling dominated by the Fgf7‐Fgfr2 axis to maintain MuSCs homeostasis. Taken together, our results demonstrate that dysregulated polyamine metabolism is a key contributor to denervation‐induced muscle atrophy, and ODC1 inhibition mitigates muscle atrophy and fibrosis by restraining proteolysis and preserving MuSCs homeostasis, which will provide references for future clinical treatments.

## Graphical abstract

Polyamine metabolism is innervation responsive and involved in denervation‐induced muscle atrophy. Inhibition of polyamine metabolism attenuates muscle atrophy by restraining proteolysis and preserving MuSCs homeostasis. Denervation‐induced activation of FAP‐derived FGF7 drives premature MuSCs activation, while DFMO suppresses this paracrine cue to preserve the MuSCs pool.

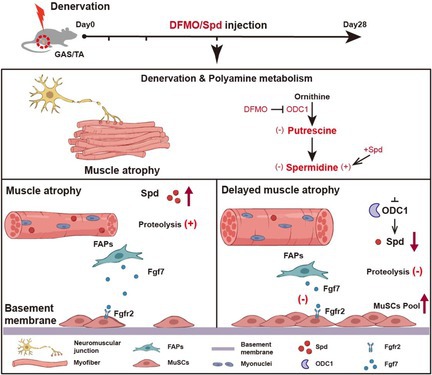

## 1 Introduction

Skeletal muscle is a highly plastic tissue that depends on intact neuromuscular innervation to maintain contractile activity, metabolic homeostasis, and structural integrity [1, 2]. Loss of neural input, as occurs after peripheral nerve injury, motor neuron disease, or prolonged immobilisation‐associated neuropathy, rapidly triggers a stereotyped program of muscle remodelling [3, 4]. This program includes muscle atrophy, activation of proteolytic systems such as the ubiquitin‐proteasome pathway and autophagy pathway, inflammatory infiltration and progressive extracellular matrix deposition that can culminate in fibrosis [5, 6]. Although denervation‐induced atrophy has been intensively studied, clinically effective interventions remain limited [7].

At the cellular level, denervation alters not only the transcriptional state of myofibers but also the behaviour of muscle stem cells (MuSCs) and stromal niche populations [8, 9]. MuSCs are essential for muscle maintenance and regeneration; however, under chronic stress they can undergo premature activation and differentiation, leading to depletion of the quiescent stem cell pool and impaired long‐term regenerative capacity [10, 11]. This balance is strongly shaped by niche‐derived cues from fibro‐adipogenic progenitors (FAPs), endothelial cells, immune cells and other interstitial populations [12]. FAPs, in particular, can provide trophic signals that support myogenesis in acute injury, but can also promote fibrogenic remodelling and maladaptive signalling depending on context [13]. Understanding how denervation reshapes both myofiber‐intrinsic programs and niche‐to‐stem‐cell communication is therefore critical for identifying actionable therapeutic targets.

Metabolic remodelling is increasingly recognised as an upstream regulator of muscle fate decisions [14]. Among metabolic pathways, the dysregulation of polyamine metabolism is the common feature of diverse muscle atrophy, such as denervation, immobilisation, cachexia and starvation [15]. Polyamine metabolism has emerged as a conserved modulator of cell growth, chromatin organisation, translation, and stress responses [16]. The major polyamines, putrescine and spermidine (Spd), are synthesised through a pathway in which ornithine decarboxylase 1 (ODC1) catalyses the first rate‐limiting step, generating putrescine that is subsequently converted to Spd by spermidine synthase (SRM) [17]. Polyamine homeostasis is tightly controlled because both deficiency and excess can perturb proteostasis and transcriptional regulation. In skeletal muscle, prior work links polyamine metabolism to myogenesis and myofiber size control [18], and pharmacological suppression of polyamine synthesis has been reported to improve muscle phenotype in amyotrophic lateral sclerosis‐induced muscle atrophy [19]. However, the polyamine species responsible for denervation‐associated pathology, and the cellular mechanisms by which polyamine modulation influences muscle atrophy remain incompletely defined.

Eflornithine (DFMO) is an irreversible inhibitor of ODC1 and an FDA‐approved drug with a well‐characterised mechanism of action [20]. Its ability to lower polyamine synthesis provides a tractable approach to test whether polyamine remodelling is causal in denervation‐induced atrophy and to dissect downstream pathways. A key unanswered question is whether denervation‐associated increases in putrescine or Spd are pathogenic, and whether therapeutic benefit arises from reducing polyamines. In parallel, it is unknown how polyamine suppression influences intercellular communication within denervated muscle.

In this study, we combined pharmacological intervention and single‐nucleus transcriptomics to define the role of polyamine metabolism in denervation‐induced muscle remodelling. We first characterised the temporal trajectory of muscle atrophy and mapped concomitant changes in polyamine content and biosynthetic enzyme expression. We then tested whether ODC1 inhibition with DFMO attenuates denervation‐induced atrophy and fibrotic remodelling and examined the contributions of individual polyamines by Spd supplementation in both healthy and denervated mice. Finally, using single‐nucleus RNA sequencing and cellchat analysis, we delineated denervation‐ and DFMO‐induced changes across myonuclear states, MuSCs populations, and niche compartments, with a focus on FAP‐driven cues that regulate MuSCs activation. Together, our work identifies Spd as a pro‐atrophic metabolite in denervated muscle and reveals that DFMO confers protection through both myofiber‐intrinsic suppression of proteolysis and niche‐mediated preservation of MuSC homeostasis, highlighting polyamine metabolism and FAP‐derived signalling as promising therapeutic targets for denervation‐associated muscle atrophy.

## 2 Materials and Methods

### 2.1 Animal Experiment

All animal experiments were approved by the Institutional Animal Care and Use Committee of Chinese PLA General Hospital. Mice were housed in specific‐pathogen‐free conditions and under the 12 h light/dark cycles, and movement and feeding were not restricted. Mice were provided by the Laboratory Animal Center of Chinese PLA General Hospital. For the experiments performed, 12‐week‐old male wild‐type mice were used.

Sciatic nerve transection procedures were as follows: the mice were anaesthetised using an intraperitoneal injection of 1% sodium pentobarbital. The sciatic nerve of the right hind limb was exposed and transected in the length of 10 mm (Den group). The sciatic nerve of the left hind limb was exposed without transection (Sham group). The mice were sacrificed at days 3, 7, 14 and 28 post denervation, and the bilateral gastrocnemius and tibialis anterior of each mouse were harvested, weighed and preserved according to the standard methods. The wet weight ratio was calculated by dividing the Den side by the Sham side.

For DFMO treatment, mice received DFMO (HY‐B0744, MCE) at 125 mg/kg body weight every other day by intraperitoneal injection. For Spd intervention, mice received Spd (S0266, Sigma) at 50 mg/kg body weight every other day by intraperitoneal injection. Control animals received intraperitoneal injection of sterile saline. For FGFR inhibitor infigratinib intervention, mice received infigratinib (HY‐13311, MCE) at 15 mg/kg body weight once daily by oral gavage.

### 2.2 Neuromuscular Junction (NMJ) Staining

NMJ morphology was assessed using whole‐mount gastrocnemius samples from sham and denervated muscle at 3 days post‐denervation. Dissected gastrocnemius was fixed in 4% paraformaldehyde, followed by permeabilisation with 0.5% Triton X‐100 for 3 h at room temperature. Postsynaptic acetylcholine receptors were labelled by incubation with CF488A‐conjugated α‐bungarotoxin (00005, Biotium, 1 μg/mL) for 2 h at room temperature. All images were captured by confocal laser scanning microscopy (Leica TCS SP5, Germany). Images were acquired in three ROIs for each muscle and each ROI contained 5–20 NMJs. The endplate area of NMJs was defined by a cluster of acetylcholine receptors, analysed by ImageJ software (NIH, USA).

### 2.3 H&E Staining, Masson‐Trichrome Staining and Sirius Red Staining

After gastrocnemius was harvested and weighed, gastrocnemius was fixed in 4% paraformaldehyde, and then dehydrated and embedded in paraffin. 4‐μm‐thick paraffin‐embedded gastrocnemius slides were obtained. H&E staining was performed according to the instructions (G1120, Solarbio). Masson‐Trichrome staining was performed according to the instructions (G1346, Solarbio). Sirius Red staining was performed according to the instructions (G1472, Solarbio).

The histological slides were captured under light microscopy. Images were acquired from at least three different and random areas for each slide. For myofiber cross‐sectional area in HE slides, single myofiber cross‐sectional area was calculated using ImageJ software (NIH, USA). The fibrotic area in Masson slides and Sirius Red slides was quantified by ImageJ software (NIH, USA).

### 2.4 Immunofluorescence Staining

The muscle sections were permeabilised and blocked with 0.3% triton X‐100 and QuickBlock blocking buffer (Beyotime) at room temperature for 30 min. Then the sections were incubated overnight at 4°C with the primary antibody, anti‐spermidine (ab7318, 1:50, Abcam), anti‐laminin (ab11575, 1:1000, Abcam), anti‐PAX7 (AB_528428, 5 μg/mL, DSHB), and anti‐MyoD (554,130, 1:500, BD) diluted in QuickBlock primary antibody dilution buffer (Beyotime). After washing 3 times, the sections were incubated for 1 h at room temperature with the secondary antibody, Alexa Fluor 647 (ab150079, 1:500, Abcam), Alexa Fluor 488 (ab150077, 1:500, Abcam), and Alexa Fluor 647 (ab150115, 1:500, Abcam) diluted in QuickBlock secondary antibody dilution buffer (Beyotime). All images were captured by confocal laser scanning microscopy (Leica TCS SP5, Germany). For spermidine staining, heat‐mediated antigen retrieval of paraffin‐embedded gastrocnemius slides was performed in Tris‐EDTA retrieval solution (pH 9.0). All images were acquired from at least three different and random areas for each slide, and each area contained 50–150 myofibers. The quantification of spermidine was based on the mean fluorescent intensity of Spd. The cross‐sectional area was quantified based on the mean area of laminin. The images were analysed by ImageJ software (NIH, USA). PAX7+ and MyoD+ cells were quantified manually using the co‐localisation of PAX7 and DAPI, or MyoD and DAPI.

### 2.5 Liquid Chromatography–Tandem Mass Spectrometry (LC–MS/MS)

Putrescine and spermidine were detected based on the AB Sciex QTRAP 6500 LC–MS/MS platform. Briefly, after the sample was thawed and smashed, an amount of 0.05 g of gastrocnemius was used to extract metabolites. The sample extracts were analysed using an LC–MS/MS system (UPLC, ExionLC AD; MS, QTRAP 6500+ System). UPLC: column, ACQUITY UPLC BEH Amide (i.d.2.1 × 100 mm, 1.7 μm); solvent system, water with 10 mM Ammonium acetate and 0.3% Ammonium hydroxide (A), 90% acetonitrile/water (V/V) (B); The gradient was started at 95% B (0–1.2 min), decreased to 70% B (8 min), 50% B (9–11 min), finally ramped back to 95% B (11.1–15 min); flow rate, 0.4 mL/min; temperature, 40°C; injection volume: 2 μL. The linear equation of standard curve for spermidine is y = 10166.05507 x + 1.23534e6 and the correlation coefficient is 0.99309. The LLOQ and ULOQ is 50 and 1000 ng/mL, respectively. The linear equation of standard curve for putrescine is y = 8754.10541 x + 5.10399e5 and the correlation coefficient is 0.99961. The LLOQ and ULOQ is 20 and 500 ng/mL, respectively.

### 2.6 RNA Extraction and Real‐Time Quantitative PCR Analysis

Total RNA was extracted from gastrocnemius samples using FastPure Cell/Tissue Total RNA Isolation Kit V2 (RC112, Vazyme Biotech), and cDNA was synthesised using the HiScript III All‐in‐one RT SuperMix Perfect for qPCR (R333, Vazyme Biotech). Then, RT‐qPCR was conducted in a CFX96 thermal cycler (Bio‐Rad) using the ChamQ Universal SYBR qPCR Master Mix (Q711, Vazyme Biotech) according to the instructions. The relative mRNA expression level was calculated by the 2^−ΔΔCt^ method, and GAPDH was used as the internal control.

The primers used were as follows: *Arg1* F: 5′‐CTCCAAGCCAAAGTCCTTAGAG‐3′, R: 5′‐AGGAGCTGTCATTAGGGACATC‐3′; *Odc1* F: 5′‐GACGAGTTTGACTGCCACATC‐3′, R: 5′‐CGCAACATAGAACGCATCCTT‐3′; *Srm* F: 5′‐GTCCAGTGCGAGATTGATGAG‐3′, R: 5′‐GAGCTGGTAATAGGACTCCTTGA‐3′; *Sms* F: 5′‐CACAGCACGCTCGACTTCAA‐3′, R: 5′‐TGCCATTCTTGTTCGTGTAAGTT‐3′; *Sat1* F: 5′‐GAGAACACCCCTTCTACCACT‐3′, R: 5′‐GCCTCTGTAATCACTCATCACGA‐3′; *Trim63* F: 5′‐AGGGCTCCCCACCACCTGTGT‐3′, R: 5′‐TGCCCTCTCTAGGCCACCG‐3′; *Fbxo32* F: 5′‐AACAAGGAGGTATACAGTAAGG‐3′, R: 5′‐AATTGTTCATGAAGTTCTTTTG‐3′; *Gapdh* F: 5′‐CAACTCCCTCAAGATTGTCAGCAA‐3′, R: 5′‐GGCATGGACTGTGGTCATGA‐3′.

### 2.7 Single‐Nucleus RNA Sequencing (snRNA‐Seq) and Data Process

Single nuclei were isolated from gastrocnemius harvested 7 days after denervation. cDNA libraries were constructed and sequenced using the DNBelab C series High‐throughput Single‐cell RNA Library Preparation Set V3.0 (TaiM 4). Sequencing of the cDNA libraries was performed on the Illumina sequencing platform by Omicsmaster Biotechnology Co. Ltd. (Guangzhou, China). For raw FASTQ files, quality control, sequence alignment, and read count quantification were conducted using dnbc4tools (version 2.1.0). Downstream analysis of the gene‐cell expression matrices were performed with Seurat (v5.0), including quality control, data integration, cell‐type annotation, differential expression analysis, gene set enrichment analysis, and cell–cell interaction analysis [21]. Low‐quality cells were excluded based on the following criteria: fewer than 400 detected genes, more than 6000 detected genes, or mitochondrial read proportion exceeding 5%. Following quality control, a total of 8321, 6768, and 6594 nuclei were retained for downstream analysis in the Con, Den, and DFMO groups, respectively, with an average of 27,000 reads and approximately 1400 genes detected in each nucleus. Highly variable genes were identified using the ‘FindVariableFeatures’ function (top 2000 features). Principal component analysis (PCA) was subsequently performed on the set of variable genes. Batch effects were corrected using the Harmony algorithm, and the top 30 Harmony components were retained for subsequent downstream analysis [22]. Specifically, the ‘RunHarmony’ function was applied with default parameters. A shared nearest neighbour (SNN) graph was constructed using ‘FindNeighbors’, followed by unsupervised clustering via the ‘FindClusters’ function, with the parameter ‘resolution = 0.2’ for the annotation of major clusters in Figure 4B. And then we performed the fine‐annotation in different lineages with the resolution parameter used as follows: muscle fibre and stem cell related nucleus in Figure 4D: 0.5, immune and stromal nucleus in Figure 5A: 0.5. For visualisation purposes, Uniform Manifold Approximation and Projection (UMAP) was performed using ‘RunUMAP’. Cell populations were annotated based on known cell‐type‐specific markers. All other parameters were kept at their default values unless otherwise specified.

### 2.8 Identification of Differentially Expressed Genes (DEGs) and Gene Set Enrichment Analysis

DEGs between groups within each cell population were identified using the ‘FindMarkers’ function in Seurat, which was based on normalised expression data and the Wilcoxon rank‐sum test. DEGs were defined using the following criteria: Benjamini‐Hochberg (BH)‐adjusted *p* value < 0.05 and |log2 fold change (log2 FC)| > 0.25. The R package clusterProfiler (version 4.14.6) was employed to perform functional annotation of DEGs and gene set enrichment analysis [23]. As for the gene set score analysis, the ‘positive regulation of autophagy’ and ‘cell activation’ gene sets were downloaded from the Molecular Signatures Database (MSigDB, https://www.gsea‐msigdb.org/gsea), while the ‘muscle atrophy’ gene set was retrieved from Daniel Taillandier et al. [24]. The ‘AddModuleScore’ function in Seurat was used to calculate gene set scores for each cell. Statistical analysis of gene set scores between groups was performed using the Wilcoxon rank‐sum test with the R package ggpubr (version 0.4.0). The R package ggplot2 (version 4.0.1) was used for visualisation of the results.

### 2.9 Cell–Cell Interaction Analysis

Ligand‐receptor interactions were detected using CellChat (v.1.1.0) on the integrated snRNA‐seq data following standard protocols [25]. The gene expression matrix and cell type annotations were imported into CellChat for analysis. Particularly, the B cell and Schwann cell clusters were excluded from the analysis due to insufficient nuclear counts. The overall communication probability among different cell clusters was calculated using the ‘computeCommunProb’ function with a trim threshold set at 0.1. A permutation test was used to verify the cellular localisation of Fgf7, Fgf5, and Fgf2 [26]. The R package ggplot2 (version 4.0.1) was used to visualise the results.

### 2.10 Cell Culture

Mouse C2C12 myoblasts were obtained from Cell Resource Center, Institute of Basic Medicine, Chinese Academy of Medical Sciences and cultured in Dulbecco's modified Eagle medium (10,569,010, Gibco) supplemented with 10% FBS (10,099,141, Gibco) and 1% Penicillin–Streptomycin (15,140,122, Gibco) under 37°C and 5% CO_2_. To induce differentiation of C2C12 cells, the growth medium was changed to differentiation medium, Dulbecco's modified Eagle medium containing 2% horse serum (26,050,088, Gibco), incubating for 5 days, when the myoblasts grew to 80%–90% confluence. To evaluate the effects of Spd or DFMO on myotube, on day 3 of differentiation, the culture medium was replaced with fresh differentiation medium supplemented with either Spd (10 μM, S0266, Sigma) or DFMO (5 mM, HY‐B0744, MCE), and cells were maintained in the corresponding treatment media for subsequent analysis.

### 2.11 Immunofluorescence Cell Staining

The differentiation ability of C2C12 cells was evaluated using MHC staining. Briefly, myotubes were fixed with 4% paraformaldehyde, permeabilised, and blocked with 0.2% Triton X‐100 and QuickBlock blocking buffer (Beyotime) at room temperature, respectively. The myotubes were incubated with primary antibody against MHC (MF20, 4 μg/mL, DSHB) overnight at 4°C, subsequently incubated with secondary antibody goat anti‐mouse Alexa Fluor 488 (ab150113, 1:500, Abcam) at room temperature. Thereafter, cells were counterstained with DAPI. Images were acquired from at least three different and random areas for each well, and each area contained 10–20 myotubes. The myotube diameter was measured as the average from three independent measurements per myotube, and only myotubes with 3 or more nuclei cells were considered. The images were analysed by ImageJ software (NIH, USA).

### 2.12 Western Blotting Analysis

GAS muscles were homogenised in RIPA lysis buffer supplemented with a protease inhibitor cocktail and the total protein content was estimated using a BCA protein concentration determination kit. Equal amounts of protein (20 μg) were separated via sodium dodecyl sulfate‐polyacrylamide gel electrophoresis (SDS‐PAGE), transferred to polyvinylidene difluoride membranes (PVDF; Bio‐Rad) using a semi‐dry transfer system (Bio‐Rad), and blocked in EveryBlot Blocking Buffer. The membranes were incubated at 4°C overnight with the following primary antibodies: anti‐MuRF1 (Abcam), anti‐Atrogin1 (Abcam), anti‐FGF7 (Abcam), and anti‐GAPDH (Abcam). The membranes were then incubated at room temperature with anti‐rabbit or anti‐mouse IgG‐conjugated secondary antibodies (Zhongshanjinqiao) for 1 h. The signal was visualised using Clarity Western ECL substrate (Bio‐Rad) and detected by a chemiluminescence imaging system (Bio‐Rad). Band grayscale was analysed using ImageJ software (NIH, US) and normalised to GAPDH.

### 2.13 Statistical Analysis

Statistical analysis was conducted using GraphPad Prism 8.0 (GraphPad) and data was presented as the mean ± standard deviation (SD). Statistical significance was evaluated by the two‐tailed unpaired Student's *t*‐test for comparing two groups and one‐way ANOVA followed by Tukey's test for comparing more than two groups. For the in vivo experiments, n represents the number of animals. For the in vitro experiments, *n* represents the number of independent experiments. *p < 0.05, **p < 0.01, ***p < 0.001, ns *p* > 0.05. *p* < 0.05 was considered statistically significant.

## 3 Results

### 3.1 Denervation Led to Dysregulated Polyamine Metabolism

**FIGURE 1:**
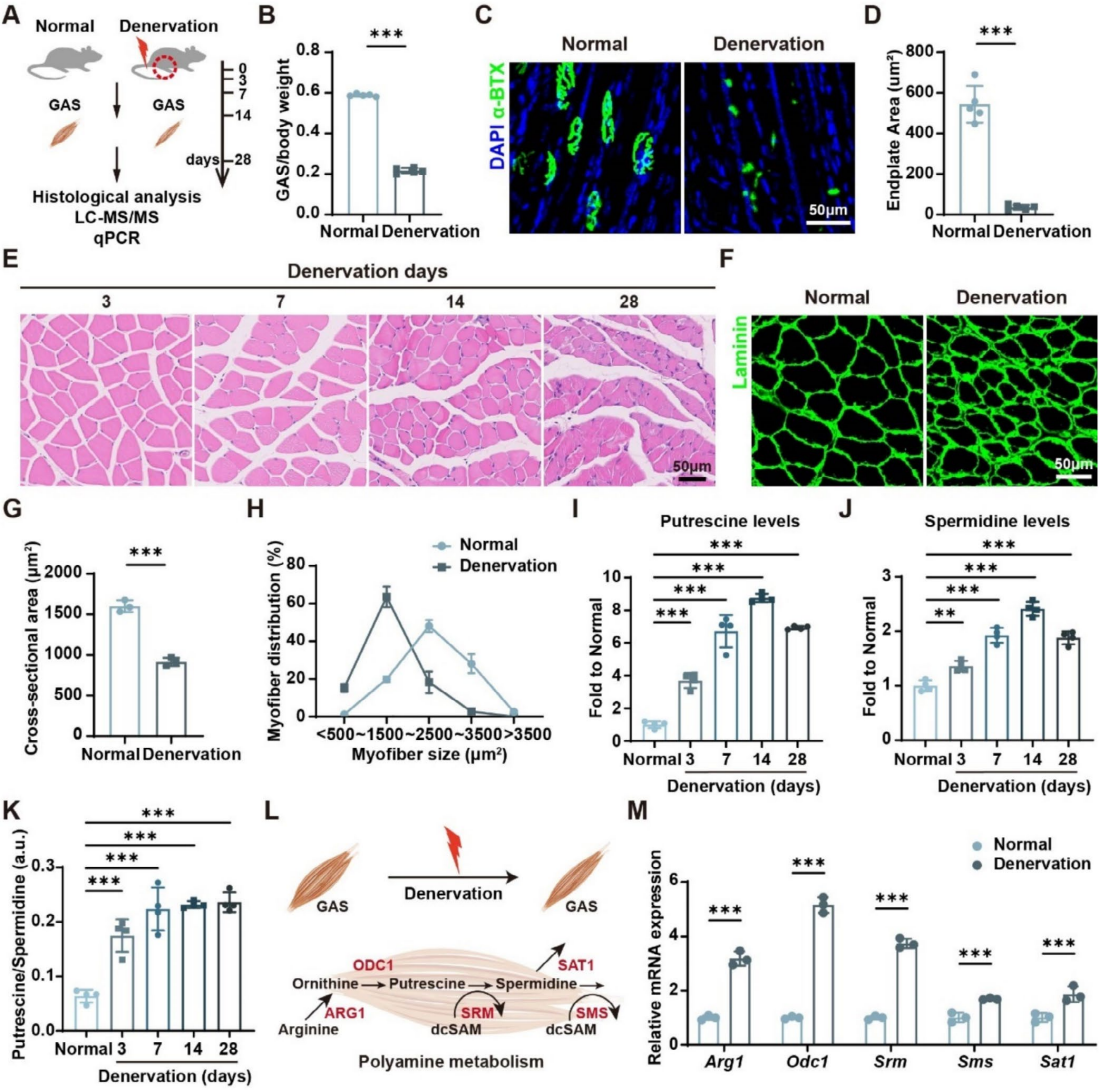
Denervation led to increased polyamine metabolism flux. (A) The schematic of the experimental design to evaluate the dynamic polyamine metabolism in denervated muscle atrophy at the indicated time points. (B) The normalised muscle weight of gastrocnemius at days 28 after denervation. (C, D) The characterisation of neuromuscular junction in gastrocnemius at days 3 after denervation. Green: α‐BTX. Blue: DAPI. Scale bar 50 μm. (E) H&E staining of cross‐sections of gastrocnemius were used to analyse histological changes after denervation. Scale bar 50 μm. (F) Representative immunofluorescence images of gastrocnemius myofiber membrane at days 14 after denervation. Green: Laminin. Scale bar 50 μm. (G, H) The cross‐sectional area and myofiber size distribution of gastrocnemius at days 14 after denervation. (I, J) LC–MS/MS quantitative analysis of putrescine and spermidine levels in denervated gastrocnemius at the indicated time points. (K) Polyamine ratio (putrescine/spermidine) in denervated gastrocnemius at the indicated time points. (L) The schematic of polyamine metabolism pathway. (M) The mRNA expression levels of polyamine metabolism enzymes, *Arg1, Odc1, Srm, Sms* and *Sat1* by RT‐qPCR in gastrocnemius at days 7 after denervation. Dots are individual values of independent animals. Data were represented as mean ± SD. Statistical tests: Two‐tailed unpaired Student's *t*‐test (B, D, G, and M), one‐way ANOVA test (I–K). **p < 0.01, ***p < 0.001 compared with Normal group. *n* = 3–5/group.

Denervation‐induced muscle atrophy occurs as an acute pathological condition distinct from the gradual loss of muscle mass observed during physiological aging. To model this process, we transected the sciatic nerve to establish a denervated muscle atrophy model (Figure 1A). Compared with normal control, denervation resulted in a marked reduction in gastrocnemius muscle weight (Figure 1B), indicating rapid loss of muscle mass. Consistently, denervated muscles exhibited degeneration of nerve terminal structures (Figure 1C,D), supporting effective disruption of neuromuscular innervation. Histopathological assessment of the gastrocnemius at multiple time points by H&E staining revealed pronounced myofiber atrophy over time, together with immune cell infiltration within the interstitial space and increased extracellular matrix deposition (Figure 1E), suggestive of an active remodelling environment following denervation. Quantitative analysis further demonstrated that denervation significantly decreased myofiber CSA (Figure 1F,G, S4B). In addition to the reduction in mean CSA, the CSA distribution was shifted toward smaller fibres (Figure 1H), reflecting a broad, population‐level atrophic response rather than changes confined to a minor subset of fibres.

Given prior evidence linking diverse atrophy states with dysregulated polyamine metabolism [15], we next profiled key polyamine metabolites using mass spectrometry. Both putrescine and Spd progressively increased after denervation, reaching maximal levels at day 14 (Figure 1I,J). This temporal pattern aligned with the observation that denervated muscle undergoes its most rapid wasting during the first two weeks after denervation. Notably, the magnitude of putrescine elevation was substantially greater than that of Spd, suggesting preferential accumulation of upstream polyamine species. Accordingly, the putrescine‐to‐spermidine ratio increased after denervation and stabilised after day 7 (Figure 1K), indicating a persistent shift in polyamine metabolic homeostasis rather than a transient fluctuation. To investigate the molecular basis of these metabolic changes, we examined the expression of enzymes governing polyamine metabolism (Figure 1L). Denervation robustly upregulated the biosynthetic enzymes arginase 1 (*Arg1*), *Odc1*, and *Srm*, with *Odc1* showing the most prominent induction, whereas catabolic enzymes, spermine synthase (*Sms*) and spermidine/spermine N(1)‐acetyltransferase 1 (*Sat1*), exhibited only modest increases (Figures 1M, S4A).

Together, these results support that denervation drives acute muscle atrophy while simultaneously promoting polyamine biosynthesis, leading to accumulation of putrescine and Spd and a sustained imbalance in polyamine metabolic flux during the early phase of denervation‐induced muscle atrophy.

### 3.2 Blocking Polyamine Biosynthesis Attenuated Denervation‐Induced Muscle Atrophy

**FIGURE 2:**
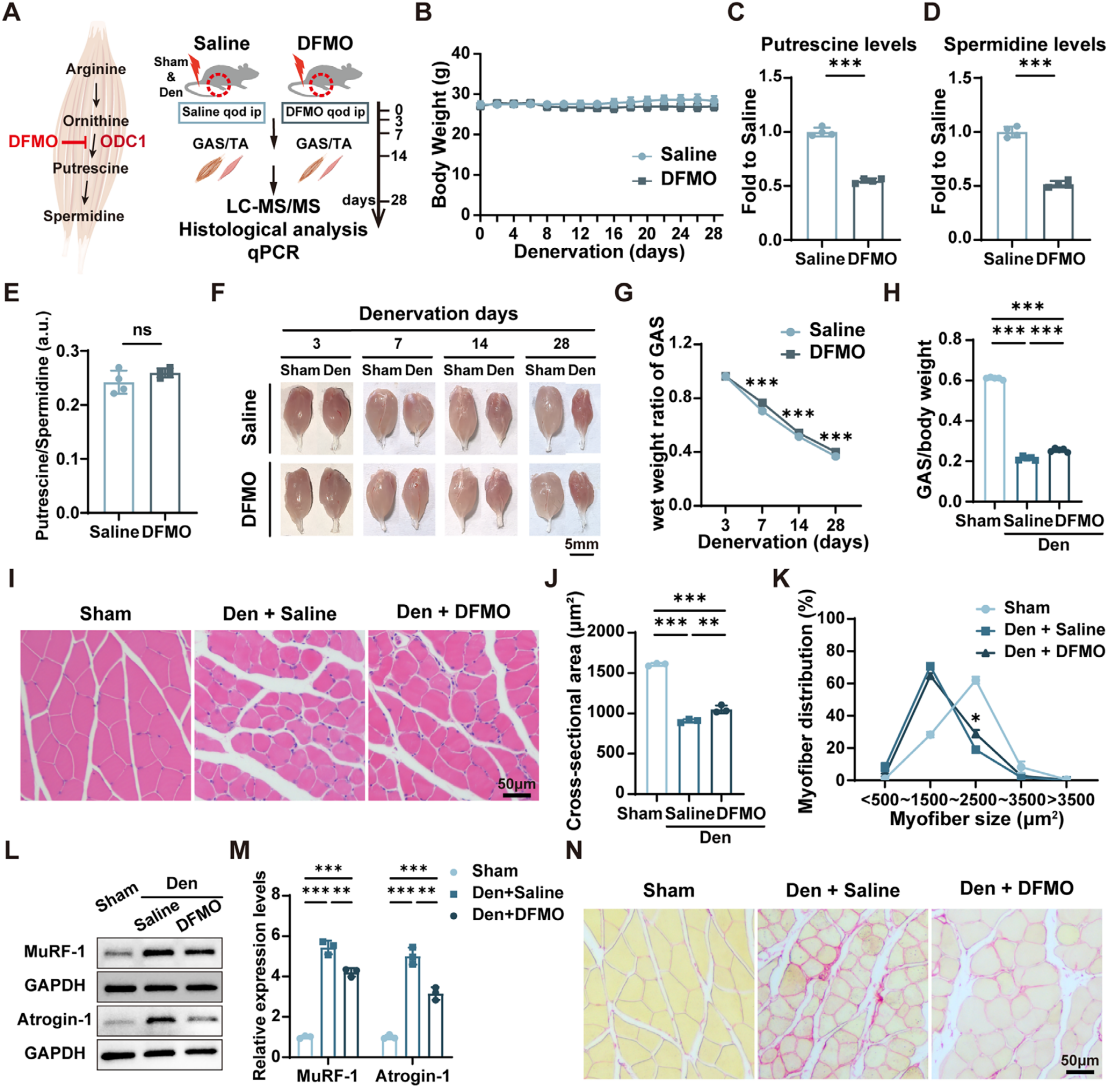
DFMO improved denervation‐induced muscle atrophy. (A) The schematic of the experimental design to evaluate the effects of DFMO in denervated muscle atrophy at the indicated time points. (B) The body weight of each mouse treated with saline or DFMO was measured for 28 days. (C, D) LC–MS/MS quantitative analysis of putrescine and spermidine levels in denervated gastrocnemius at days 7 after treated with saline or DFMO. (E) Polyamine ratio (putrescine/spermidine) in denervated gastrocnemius at days 7 after treated with saline or DFMO. (F) Gross morphology of gastrocnemius harvested from different treatment groups at days 3, 7, 14 and 28 after denervation. Scale bar 5 mm. (G) The wet weight ratio of gastrocnemius at the indicated time points after denervation. (H) The normalised muscle weight of gastrocnemius at days 28 after denervation treated with saline or DFMO. (I) H&E staining of cross‐sections of gastrocnemius were used to analyse cross‐sectional area at days 14 after denervation treated with saline or DFMO. Scale bar 50 μm. (J, K) The cross‐sectional area and myofiber size distribution of gastrocnemius at days 14 after denervation treated with saline or DFMO. (L, M) The expressions of E3 ubiquitin ligases MuRF‐1 and Atrogin‐1 quantified via western blotting in gastrocnemius at days 7 after denervation treated with saline or DFMO. (N) The representative Sirius Red staining images of gastrocnemius at days 14 after denervation treated with saline or DFMO. Scale bar 50 μm. Dots are individual values of independent animals. Data were represented as mean ± SD. Statistical tests: Two‐tailed unpaired Student's *t*‐test (C–E and G), one‐way ANOVA test (H, J, K, and M). ns *p* > 0.05, * *p* < 0.05, **p < 0.01, ***p < 0.001. *n* = 3–5/group.

Given the marked increase in polyamine biosynthetic flux after denervation, we next asked whether pharmacological blockade of this pathway could mitigate denervation‐induced muscle atrophy. consequently, mice received intraperitoneal injections of the ODC1 inhibitor DFMO, an FDA‐approved drug for neuroblastoma and trypanosomiasis (Figure 2A). DFMO was administered for 28 days and was well tolerated, as evidenced by unchanged body weight relative to saline‐treated controls (Figure 2B). We first evaluated the polyamine metabolites by mass spectrometry. DFMO significantly reduced both putrescine and Spd levels in denervated muscles (Figure 2C,D). Notably, Spd content was restored to a level comparable to that of healthy muscle, whereas putrescine, although substantially decreased, remained elevated compared with normal control. Despite these changes in absolute metabolite abundance, the putrescine‐to‐spermidine ratio showed no significant difference between DFMO and saline groups (Figure 2E), suggesting that DFMO primarily reduced pathway flux rather than inducing a sustained shift in relative polyamine composition.

We next assessed whether polyamine suppression could improve muscle atrophy. Gross morphology of gastrocnemius and tibialis anterior showed an attenuated atrophic appearance in DFMO‐treated mice (Figures 2F, S1A). Quantitatively, gastrocnemius and tibialis anterior wet weight ratios were higher in the DFMO group than in the Saline group (Figures 2G, S1B), and the normalised muscle weight showed the same protective trend (Figures 2H, S1C), indicating that DFMO delayed the progression of denervation‐induced muscle loss. Histological evaluation by H&E staining further supported that DFMO increased mean CSA and shifted the CSA distribution toward larger myofibers (Figure 2I–K), indicating improved muscle atrophy. Activation of proteolytic programs is a hallmark driver of denervated muscle atrophy [27]. We further examined the ubiquitin‐proteasome pathway by measuring MuRF‐1 (*Trim63*) and Atrogin‐1 (*Fbxo32*). Denervation robustly induced the expression of MuRF‐1 (*Trim63*) and Atrogin‐1 (*Fbxo32*), whereas DFMO treatment blunted this induction, consistent with reduced activation of catabolic signalling (Figures 2L,M, S1D, and S4A). In addition to muscle atrophy, denervation induced progressive accumulation of fibrosis that can compromise contractile function via excessive extracellular matrix deposition [28]. Sirius Red and Masson's trichrome staining revealed that denervation markedly increased collagen accumulation and expanded the fibrotic area, while DFMO significantly alleviated these fibrotic changes (Figures 2N, S1E–G).

Collectively, these results demonstrate that ODC1 inhibition by DFMO effectively suppresses polyamine accumulation in denervated muscle and concomitantly attenuates muscle atrophy, reduces proteasome‐associated atrogene activation, and improves the fibrosis phenotype. These findings identify polyamine biosynthesis as a mechanistically relevant and therapeutically actionable axis in denervation‐induced muscle atrophy.

### 3.3 Spd Treatment Promoted Muscle Atrophy in Normal and Denervation Conditions

**FIGURE 3:**
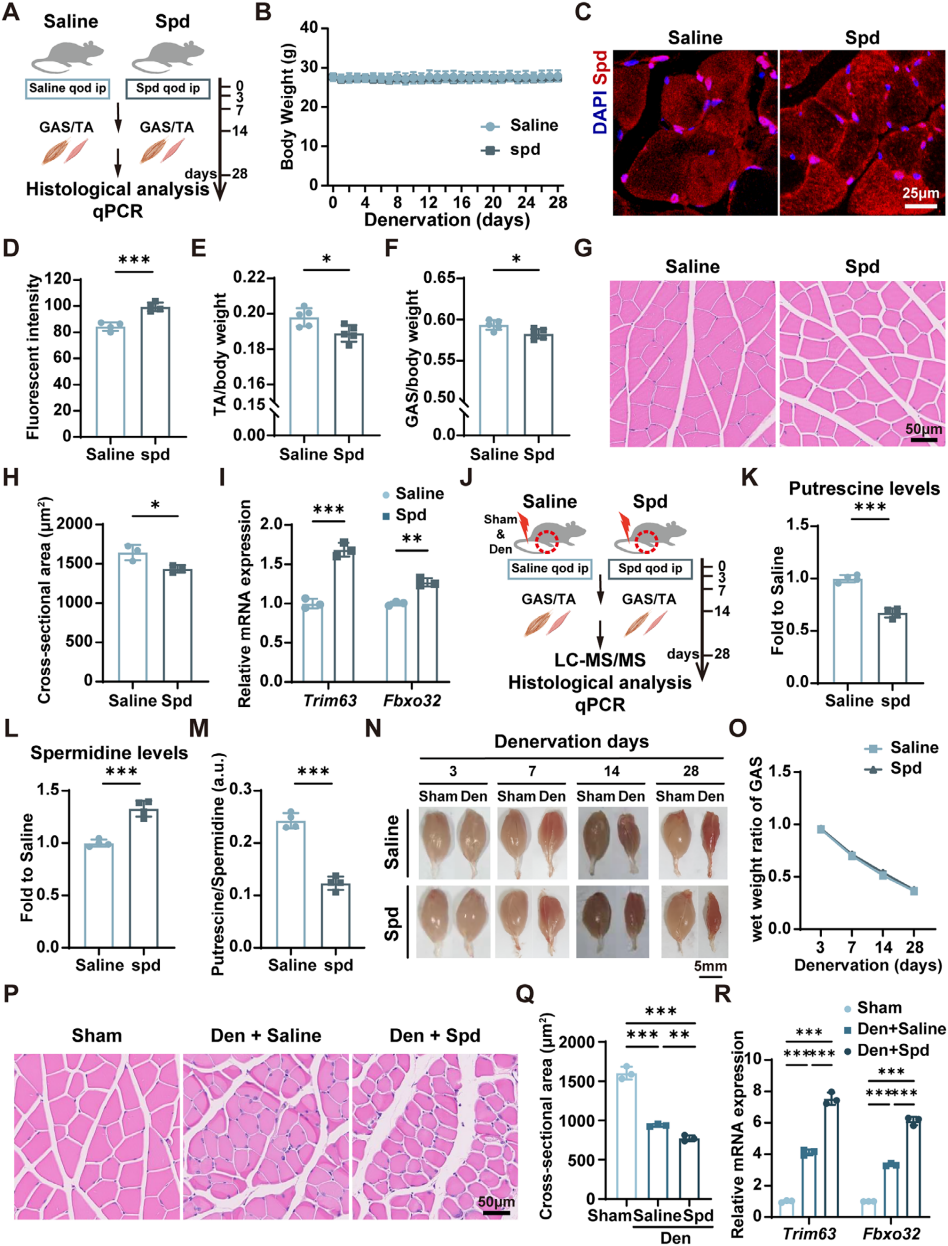
Spd treatment elicited slight muscle atrophy in healthy muscle and aggravated denervation‐induced muscle atrophy. (A) The schematic of the experimental design to evaluate the effects of spermidine in young mice at the indicated time points. (B) The body weight of each mouse treated with saline or spermidine was measured for consecutive 28 days. (C, D) Representative immunofluorescence images and quantification of fluorescent intensity of spermidine in gastrocnemius treated with saline or spermidine. Red: Spermidine. Blue: DAPI. Scale bar 50 μm. (E, F) The normalised muscle weight of tibialis anterior and gastrocnemius at days 28 after treated with saline or spermidine. (G, H) H&E staining of cross‐sections of gastrocnemius were used to analyse cross‐sectional area at days 28 after treated with saline or spermidine. Scale bar 50 μm. (I) The mRNA expression levels of E3 ubiquitin ligases, *Trim63* and *Fbxo32*, by RT‐qPCR in gastrocnemius muscles after treated with saline or spermidine. (J) The schematic of the experimental design to evaluate the effects of spermidine in denervated muscle atrophy at the indicated time points. (K, L) LC–MS/MS quantitative analysis of putrescine and spermidine levels in denervated gastrocnemius at days 7 after treated with saline or spermidine. (M) Polyamine ratio (putrescine/spermidine) in denervated gastrocnemius at days 7 after treated with saline or spermidine. (N) Gross morphology of gastrocnemius harvested from different treatment groups at days 3, 7, 14 and 28 after denervation. Scale bar 5 mm. (O) The wet weight ratio of gastrocnemius at the indicated time points after denervation. (P) H&E staining of cross‐sections of gastrocnemius were used to analyse cross‐sectional area at days 14 after denervation treated with saline or spermidine. Scale bar 50 μm. (Q) The cross‐sectional area of gastrocnemius at days 14 after denervation treated with saline or spermidine. (R) The mRNA expression levels of E3 ubiquitin ligases, *Trim63* and *Fbxo32*, by RT‐qPCR in gastrocnemius post denervation. Dots are individual values of independent animals. Data were represented as mean ± SD. Statistical tests: Two‐tailed unpaired Student's *t*‐test (D–F, H, I, K–M, and O), one‐way ANOVA test (Q and R). *p < 0.05, **p < 0.01, ***p < 0.001. *n* = 3–5/group.

Considering that blocking polyamine biosynthesis alleviated denervation‐induced muscle atrophy, we next sought to delineate which elevated polyamine species contributed to the atrophic phenotype. Notably, under DFMO treatment Spd was reduced to levels comparable to healthy muscle whereas putrescine remained higher than baseline, arguing against putrescine accumulation as the primary driver of atrophy by virtue of the beneficial effects of DFMO. Previous reports showed that putrescine can promote myotube hypertrophy, whereas Spd promoted myotube atrophy [15], suggesting that the elevation of putrescine may represent a compensatory beneficial response. We therefore focused on Spd and assessed its effects in healthy young mice (Figure 3A). Intraperitoneal Spd administration did not significantly alter body weight, indicating minimal systemic impact (Figure 3B). Spd immunofluorescence in skeletal muscle confirmed increased intramuscular Spd content, with signal detected in both myofibers and myonuclei (Figures 3C,D, S4C), consistent with a potential role in regulating transcriptional programs. Further, Spd supplementation induced a modest reduction in tibialis anterior and gastrocnemius mass (Figure 3E,F). Morphometric analysis revealed a concomitant decrease in myofiber CSA (Figure 3G,H), supporting the occurrence of muscle atrophy. In line with this phenotype, Spd increased expression of the ubiquitin‐proteasome atrogenes *Trim63* and *Fbxo32* (Figures 3I, S4A), indicating enhanced protein degradation.

We next examined whether Spd also modulated muscle loss in the denervation setting (Figure 3J). Denervated mice receiving Spd exhibited a clear shift in polyamine profiles by mass spectrometry. Putrescine levels were reduced, Spd levels were increased, and the putrescine‐to‐spermidine ratio decreased (Figure 3K–M), confirming effective remodelling of polyamine composition. At the gross level, gastrocnemius and tibialis anterior wet weight ratios were not significantly different between Spd‐ and saline‐treated groups (Figures 3N,O, S2A,B), which may reflect parallel atrophy in both sham and denervated muscles under Spd treatment, thereby masking relative differences in weight‐based metrics. However, histopathological quantification confirmed that Spd significantly reduced myofiber CSA in denervated muscle (Figures 3P,Q, S2C), demonstrating that Spd exacerbated denervation‐induced muscle atrophy. Mechanistically, Spd further amplified the denervation‐driven induction of MuRF‐1 (*Trim63*) and *Fbxo32* (Figures 3R, S2D,E, and S4A), consistent with heightened activation of the ubiquitin‐proteasome pathway. By contrast, assessment of fibrosis showed no significant effect of Spd on denervation‐associated collagen deposition (Figure S2F–I), suggesting that Spd preferentially influences the proteolysis axis rather than extracellular matrix remodelling in this model.

Together, these results indicate that Spd promotes muscle atrophy in both healthy and denervated conditions, providing a plausible explanation for why DFMO‐mediated reduction of Spd associates with improved muscle atrophy.

### 3.4 Blocking Polyamine Biosynthesis Attenuates Proteolysis and Preserves MuSCs Homeostasis Revealed by snRNA‐Seq

**FIGURE 4:**
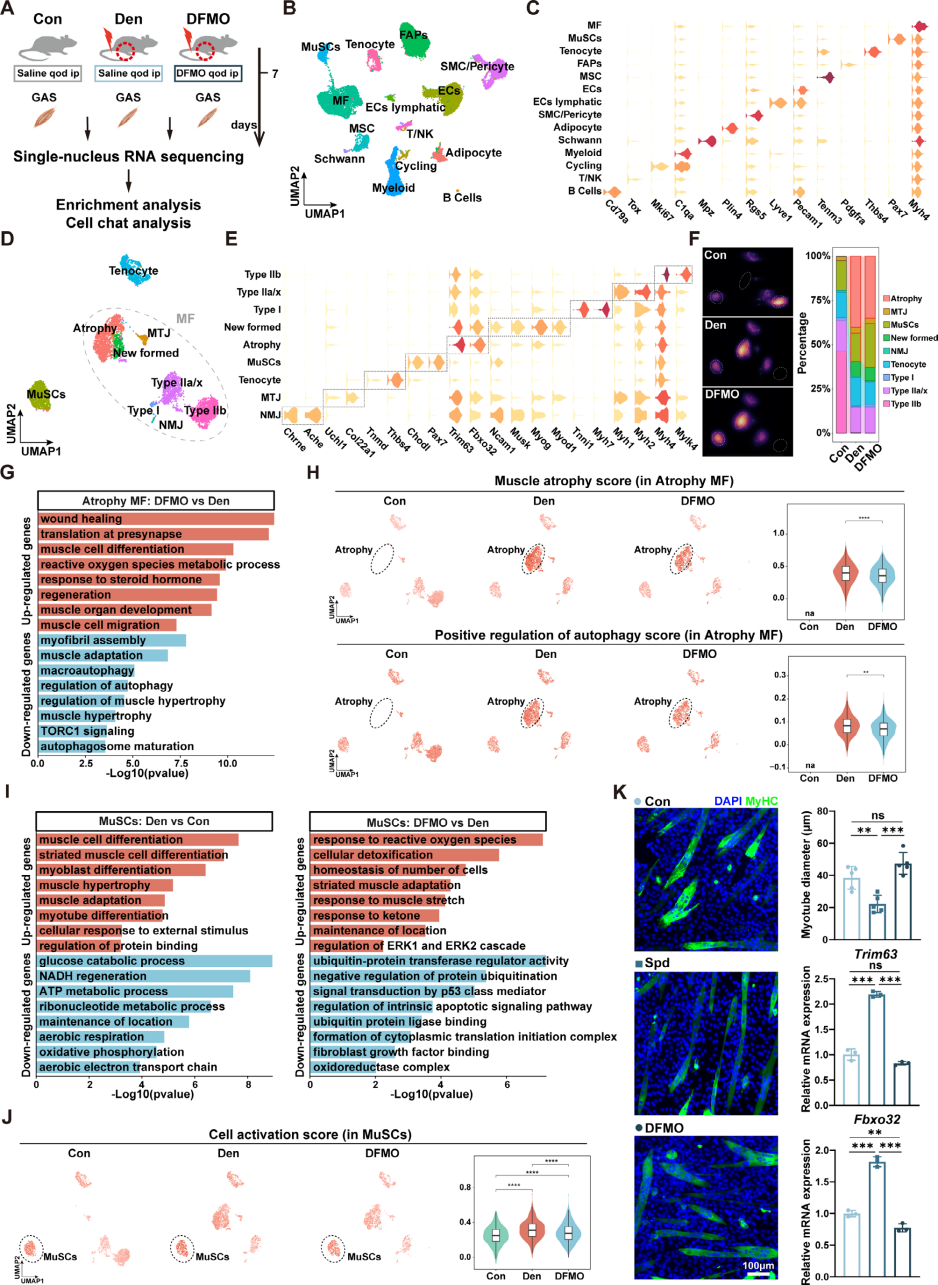
DFMO treatment inhibited proteolysis and maintained MuSCs homeostasis post denervation. (A) The schematic of the experimental design to evaluate the effects of DFMO in muscle atrophy at days 7 after denervation by single‐nucleus sequencing. (B) UMAP plot labelled by 14 distinct cell types of gastrocnemius. (C) Violin plot showing differently expressed marker genes associated with 14 distinct cell types of gastrocnemius. (D) UMAP plot labelled by 9 distinct nuclei from MF, MuSCs and tenocyte in gastrocnemius. (E) Violin plot showing differently expressed marker genes associated with 9 distinct nuclei from MF, MuSCs and tenocyte in gastrocnemius. (F) Galaxy plots (left) depicting the cell density in UMAP space for all cell types from Con (top), Den (middle), and DFMO (bottom) group mice. Cooler colours indicate low density, and warmer colours indicate high density. The relative proportion of these cell types is shown in stacked bar plots (right), and the colour indicates the different cell types. The cell types with significant changes in cell proportion are marked with white dashed circles in galaxy plots to indicate their corresponding positions. (G) Gene Ontology enrichment analysis of differentially expressed genes in atrophic myonuclei comparing DFMO‐treated vs. denervated muscles. (H) UMAP plots showing the muscle atrophy score and positive regulation of autophagy score in atrophic myonuclei cluster (dashed outline) and violin plots quantifying the muscle atrophy score and positive regulation of autophagy score across the three groups. (I) Gene Ontology enrichment analysis of differentially expressed genes in MuSCs comparing denervated and control muscles and DFMO‐treated vs. denervated muscles. (J) UMAP plots showing the cell activation score in MuSCs (dashed outline) and violin plots quantifying the cell activation score across the three groups. (K) Representative immunofluorescence images of MyHC‐stained myotubes at days 5 after the induction of differentiation. Green: MyHC. Blue: DAPI. Scale bar 100 μm. The myotube diameter to evaluate the effect of spermidine and DFMO on myotube. The mRNA expression levels of E3 ubiquitin ligases, *Trim63* and *Fbxo32*, by RT‐qPCR in differentiated myotube. *n* = 3–5/group. Dots are individual values of independent experiments (K). Data were represented as mean ± SD. Statistical tests: One‐way ANOVA test (K). ns *p* > 0.05, **p < 0.01, ***p < 0.001.

To further elucidate how inhibition of the polyamine pathway alleviates denervation‐induced muscle atrophy, we performed single‐nucleus RNA sequencing in the gastrocnemius from three conditions, sham (Con), 7 days post‐denervation (Den), and denervated mice treated with DFMO for 7 days (DFMO) (Figure 4A). Unsupervised clustering and annotation based on canonical marker genes identified 14 nuclear populations, including myofibers (MF; Myh4), MuSCs (Pax7), tenocytes (Thbs4), and FAPs (Pdgfra), among others (Figure 4B,C). Comparative analysis across cell types revealed that denervation induced prominent transcriptional remodelling in endothelial cells, FAPs, myofibers, and myeloid cells, indicating broad niche‐level responses to the loss of innervation (Figure S3A,B). We first focused on myofibers. Differential expression followed by Gene Ontology (GO) enrichment demonstrated that, relative to Con, Den myofibers displayed marked upregulation of pathways related to macroautophagy, proteolysis, neuron apoptotic process, and muscle cell differentiation, accompanied by downregulation of cellular respiration, electron transport chain, and muscle contraction, consistent with enhanced catabolism, impaired bioenergetics, and reduced contractile function during denervation while concomitantly engaging repair‐related programs (Figure S3C). In DFMO‐treated denervated muscles, myofiber GO terms shifted toward increased wound healing, myoblast differentiation, and ERK‐associated signalling, whereas autophagy and oxidative phosphorylation pathways were reduced compared with Den (Figure S3C). These pathway‐level changes aligned with our phenotypic observations, supporting the notion that DFMO attenuates denervation‐driven catabolic remodelling.

Because denervation is known to strongly reprogram myonuclear transcription and atrophy preferentially affects type IIb myofibers [8, 29], we performed refined subclustering of myofiber‐associated nuclei (Figure 4D). In addition to expected nuclei corresponding to type I, IIa, IIx, and IIb myofibers as well as MTJ‐ and NMJ‐associated nuclei and MuSCs, we identified two additional states, an atrophic myonuclear population characterised by high *Trim63* and *Fbxo32* expression, and a newly formed myonuclear population expressing *Myod1* and *Myog* (Figure 4D,E), suggestive of ongoing myogenic remodelling. Quantification of population proportions revealed that denervation was associated with a pronounced loss of type IIb nuclei, accompanied by significant expansion of atrophic and newly formed myonuclei (Figure 4F). Notably, DFMO treatment reduced the fraction of atrophic myonuclei, paralleling improved atrophy phenotypes, and increased the abundance of MuSCs compared with Den (Figure 4F), implicating both myofiber‐intrinsic and stem cell‐related mechanisms. We next interrogated transcriptional changes specifically within atrophic myonuclei. Compared with Den, DFMO altered the atrophy‐associated program by enriching GO terms related to wound healing, muscle cell differentiation, muscle organ development and regeneration while suppressing autophagy‐related pathways (Figure 4G). In parallel, module scoring showed that both muscle atrophy and positive regulation of autophagy signatures were significantly reduced in atrophic myonuclei following DFMO, supporting that DFMO mitigates proteolytic loss partly through dampening autophagy (Figure 4H). Given that Spd is a recognised autophagy inducer [30], we infer that DFMO‐mediated reduction of Spd contributes to decreased autophagic flux and consequent reduction in protein breakdown during denervation.

In MuSCs, denervation induced a transcriptional shift consistent with premature activation and differentiation [31]. Compared with Con, Den MuSCs upregulated muscle cell differentiation and muscle hypertrophy‐associated programs while downregulating glucose catabolic processes and aerobic respiration pathways (Figure 4I), indicating rapid commitment in response to denervation at the expense of metabolic homeostasis. DFMO treatment upregulated pathways linked to cell number homeostasis and the regulation of ERK signalling, while reducing ubiquitination‐ and apoptosis‐regulatory programs (Figure 4I). Consistent with the observed increase in MuSCs abundance in DFMO‐treated muscles, the cell activation gene score rose after denervation but was significantly decreased with DFMO (Figure 4J), indicating that DFMO restrains denervation‐induced MuSCs hyperactivation and helps preserve the stem cell pool. Finally, we validated key observations in vitro using differentiated myotubes. Spd treatment induced myotube atrophy, whereas DFMO did not reduce myotube diameter (Figures 4K, S4D). At the molecular level, Spd increased the expression of the atrophy markers *Trim63* and *Fbxo32*, while DFMO suppressed the expression (Figures 4K, S4A).

Together, our results suggest that DFMO lowers Spd levels, limits autophagy‐associated catabolism, preserves MuSCs homeostasis, and thereby produces a sustained improvement in the denervation‐induced muscle atrophy.

### 3.5 DFMO Reprogramed the Muscle Niche and Suppressed FAPs‐Derived Fgf7‐Fgfr2 Signalling to Preserve MuSCs Homeostasis

**FIGURE 5:**
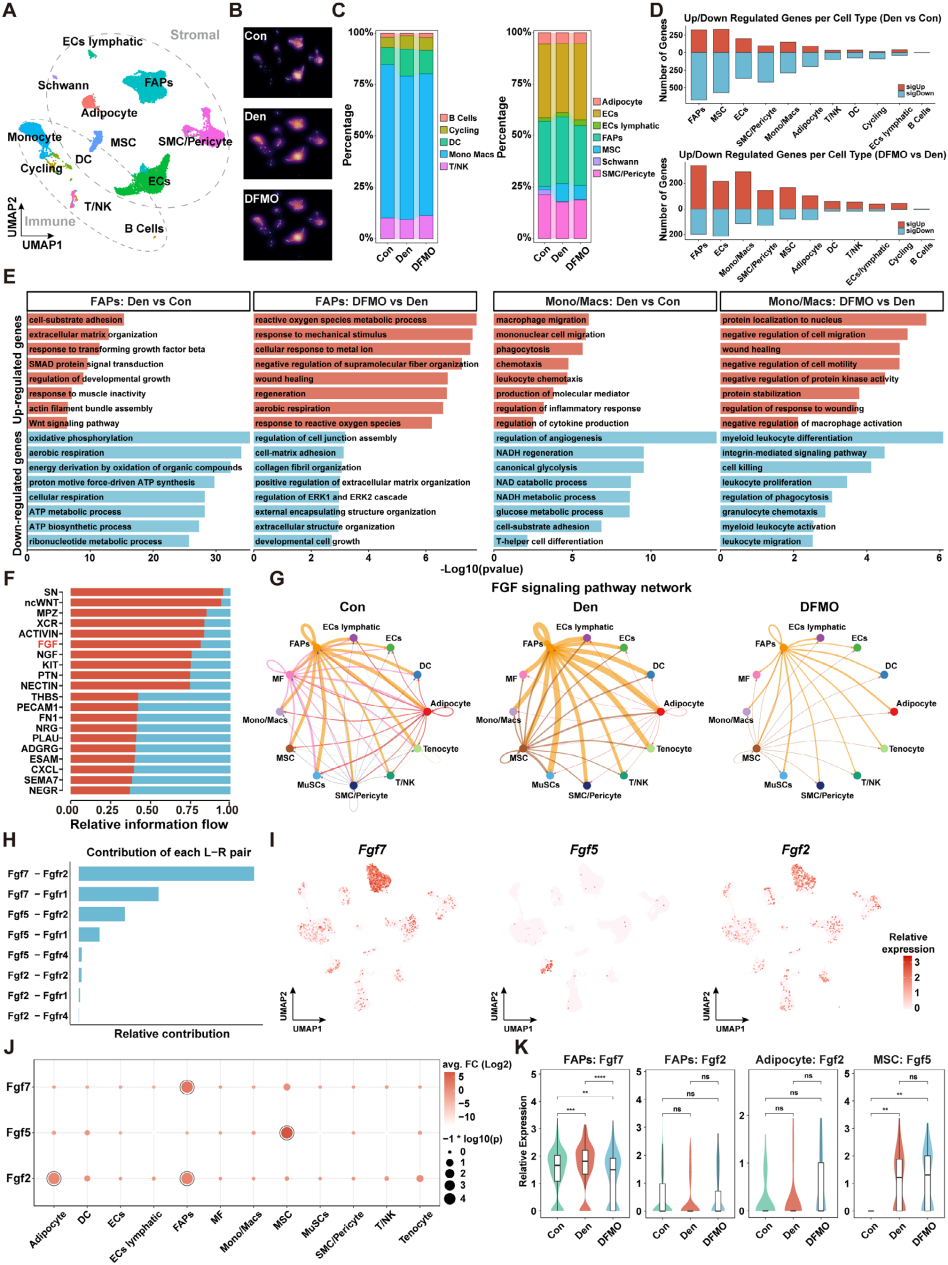
DFMO suppressed denervation‐enhanced FGF signalling. (A) UMAP plot labelled by 11 distinct cell types from immune and stromal cells in gastrocnemius. (B) The cell density in UMAP space for stromal and immune cell types from Con (top), Den (middle), and DFMO (bottom) group mice are shown in galaxy plots. The cooler colours represent low density, and warmer colours indicate high density. (C) Stacked bar plots depicting the relative proportion of each annotated population across the three groups. (D) Stacked bar plots summarise the number of significantly upregulated and downregulated genes for each annotated cell type. Top, Den vs. Con; bottom, DFMO vs. Den. (E) Gene Ontology enrichment analysis of differentially expressed genes in FAPs and Mono/Macs comparing denervated and control muscles and DFMO‐treated vs. denervated muscles. (F) Horizontal stacked bars showing the relative information flow for inferred signalling pathways based on ligand‐receptor communication analysis. (G) Cell–cell communication network analysis of the FGF signalling pathway across three groups. (H) Relative contribution of individual ligand‐receptor pair to the overall FGF signalling pathway. (I) Feature plots showing single‐nucleus expression patterns of *Fgf7*, *Fgf5* and *Fgf2* on the UMAP embedding. (J) Dot plot summarising expression of *Fgf7*, *Fgf5* and *Fgf2* across annotated cell types. (K) Violin plots comparing *Fgf7* and Fgf2 expression in FAPs, *Fgf2* expression in adipocytes, and *Fgf5* expression in MSCs across three groups.

Given the pivotal role of niche cells in regulating both myofibers and MuSCs' behaviour, we next investigated how denervation and DFMO reshape the muscle microenvironment. We grouped niche populations into immune and stromal compartments (Figure 5A). Overall, denervation caused only modest shifts in the relative abundance of immune cells, whereas stromal populations exhibited more pronounced compositional changes, particularly within endothelial cells, MSCs, and FAPs (Figure 5B,C). Consistent with this, quantification of differentially expressed genes across populations indicated that FAPs represented the most transcriptionally responsive stromal cell type after denervation and remained the most affected stromal population following DFMO treatment (Figure 5D). Among immune populations, monocytes/macrophages showed the strongest DFMO‐associated transcriptional changes (Figure 5D). Consequently, we further focused on FAPs and monocytes/macrophages.

In FAPs, denervation induced a marked functional and metabolic reprogramming. Compared with Con, Den FAPs were enriched for extracellular matrix organisation, response to transforming growth factor beta and response to muscle inactivity, while oxidative phosphorylation and cellular respiration terms were significantly downregulated (Figure 5E), suggesting a shift away from oxidative metabolism toward an activated, matrix‐remodelling state. Importantly, DFMO treatment upregulated wound healing and regeneration‐associated pathways, whereas collagen fibril organisation and extracellular matrix organisation pathways were downregulated (Figure 5E), mirroring the reduced fibrotic remodelling observed at the tissue level. These results indicate that DFMO not only acts on myofiber‐associated programmes but also rebalances stromal remodelling dynamics by restraining pro‐fibrotic FAPs activation.

Monocytes/macrophages also displayed robust denervation‐induced inflammatory activation [32]. Relative to Con, Den monocytes/macrophages showed upregulation of gene programs related to macrophage migration, chemotaxis, phagocytosis, inflammatory responses and cytokine production (Figure 5E). Following DFMO treatment, macrophage transcriptional signatures shifted toward resolution. Terms associated with negative regulation of cell migration, wound healing and negative regulation of macrophage activation were enriched, whereas leukocyte differentiation and proliferation, regulation of phagocytosis and leukocyte activation pathways were reduced (Figure 5E). Together, these findings support that DFMO may alleviate denervated muscle atrophy by dampening inflammatory activation and promoting a more pro‐repair immune microenvironment.

To mechanistically connect niche remodelling with MuSCs regulation, we performed cell–cell communication analysis. FGF signalling emerged as a prominently activated pathway after denervation and was markedly suppressed by DFMO (Figure 5F,G). FAPs were identified as the major source of FGF ligands, and the inferred signalling from FAPs to MuSCs was concomitantly reduced after DFMO treatment (Figure 5G). Dissection of ligand‐receptor contributions highlighted Fgf7‐Fgfr2 as the dominant pair within the FGF signalling network (Figure 5H). Consistent with this, Fgf7 expression was enriched in FAPs and decreased with DFMO, whereas Fgf5 (primarily expressed by MSCs) showed little change and Fgf2 (expressed by adipocytes and FAPs) remained largely comparable across groups (Figure 5I–K). Given prior evidence that the FGF7‐FGFR2 axis can promote MuSCs proliferation and differentiation [33], our data support that denervation‐induced activation of FAP‐derived FGF7 drives premature MuSCs activation, while DFMO suppresses this paracrine cue, helping preserve the MuSCs pool and ultimately improving the muscle atrophy phenotype.

**FIGURE 6:**
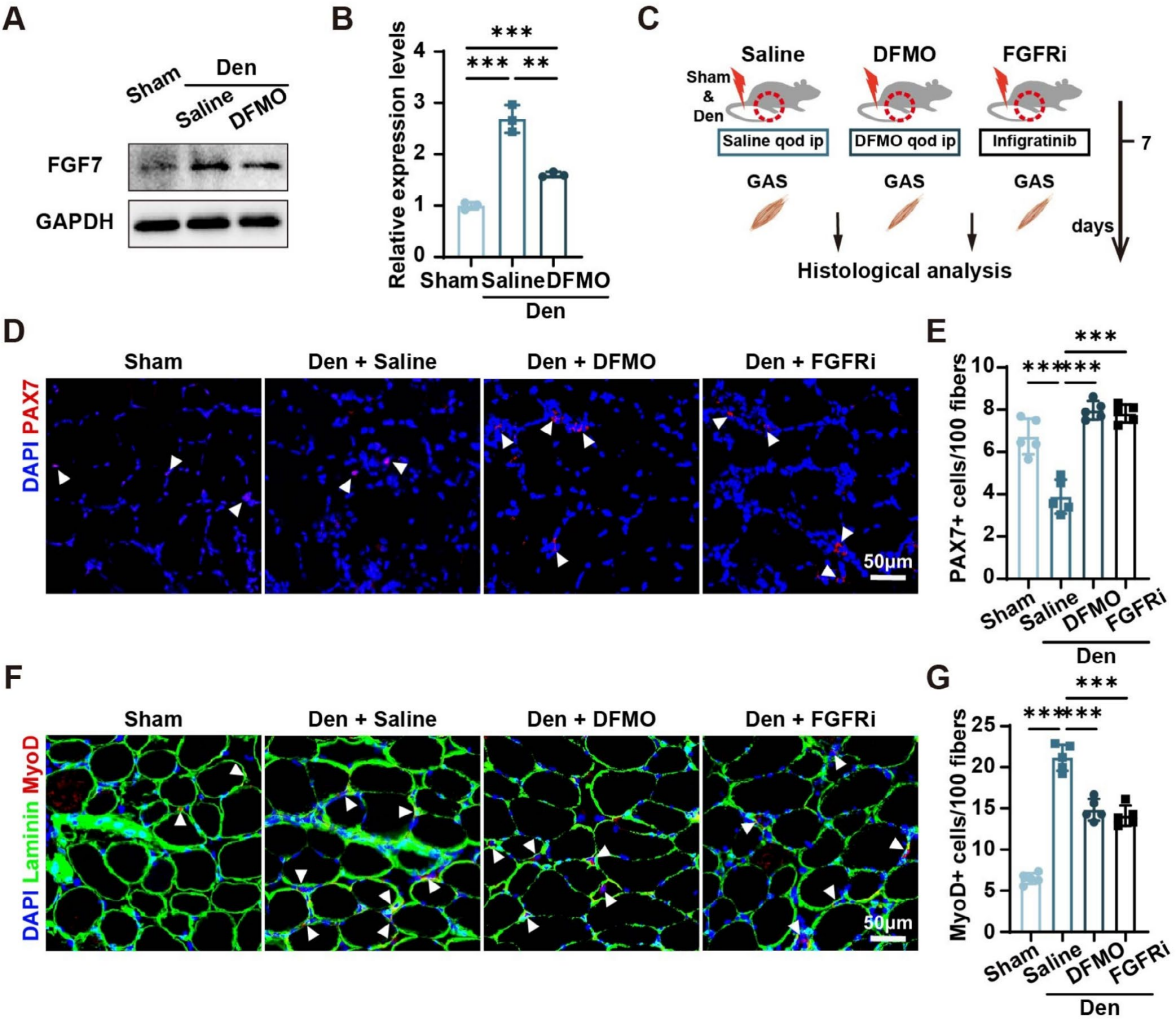
DFMO and FGFR inhibitor infigratinib treatment maintained MuSCs homeostasis post denervation. (A, B) The expression of FGF7 quantified via western blotting in gastrocnemius at days 7 after denervation treated with saline or DFMO. (C) The schematic of the experimental design to evaluate the effects of DFMO and FGFRi infigratinib on MuSCs in denervated muscle atrophy at days 7 after denervation. (D, E) Representative immunofluorescence images of PAX7 staining and quantification of PAX7+ cells in gastrocnemius treated with saline, DFMO or FGFRi. Red: PAX7. Blue: DAPI. Scale bar 50 μm. (F, G) Representative immunofluorescence images of MyoD staining and quantification of MyoD+ cells in gastrocnemius treated with saline, DFMO or FGFRi. Red: MyoD. Green: Laminin. Blue: DAPI. Scale bar 50 μm. Dots are individual values of independent animals. Data were represented as mean ± SD. Statistical tests: One‐way ANOVA test (B, E, and G). **p < 0.01, ***p < 0.001. *n* = 3–5/group.

We further performed validation by detecting the FGF7 protein expression. We observed that FGF7 expression was increased in denervated muscle and significantly reduced by DFMO treatment (Figure 6A,B), in agreement with the transcriptomic and cell communication analysis. To assess the role of FGF7‐FGFR2 signalling in MuSCs homeostasis after denervation, we pharmacologically inhibited FGFR activity using the pan‐FGFR inhibitor infigratinib (Figure 6C). PAX7 staining showed that denervation reduced the satellite cell pool, whereas both DFMO and infigratinib treatment increased the number of PAX7+ satellite cells (Figure 6D,E), indicating improved preservation of the satellite cell pool. We further evaluated MuSCs activation status and found that denervation markedly induced MuSCs activation, while both DFMO and infigratinib treatment suppressed MuSCs activation (Figure 6F,G), thereby maintaining MuSCs homeostasis.

Together, Spd reduction by DFMO likely exerts both direct effects on MuSCs states and indirect niche‐mediated regulation via FAPs, nominating the FGF7‐FGFR2 axis as a potential therapeutic target in denervation‐induced muscle atrophy.

## 4 Discussion

**FIGURE 7:**
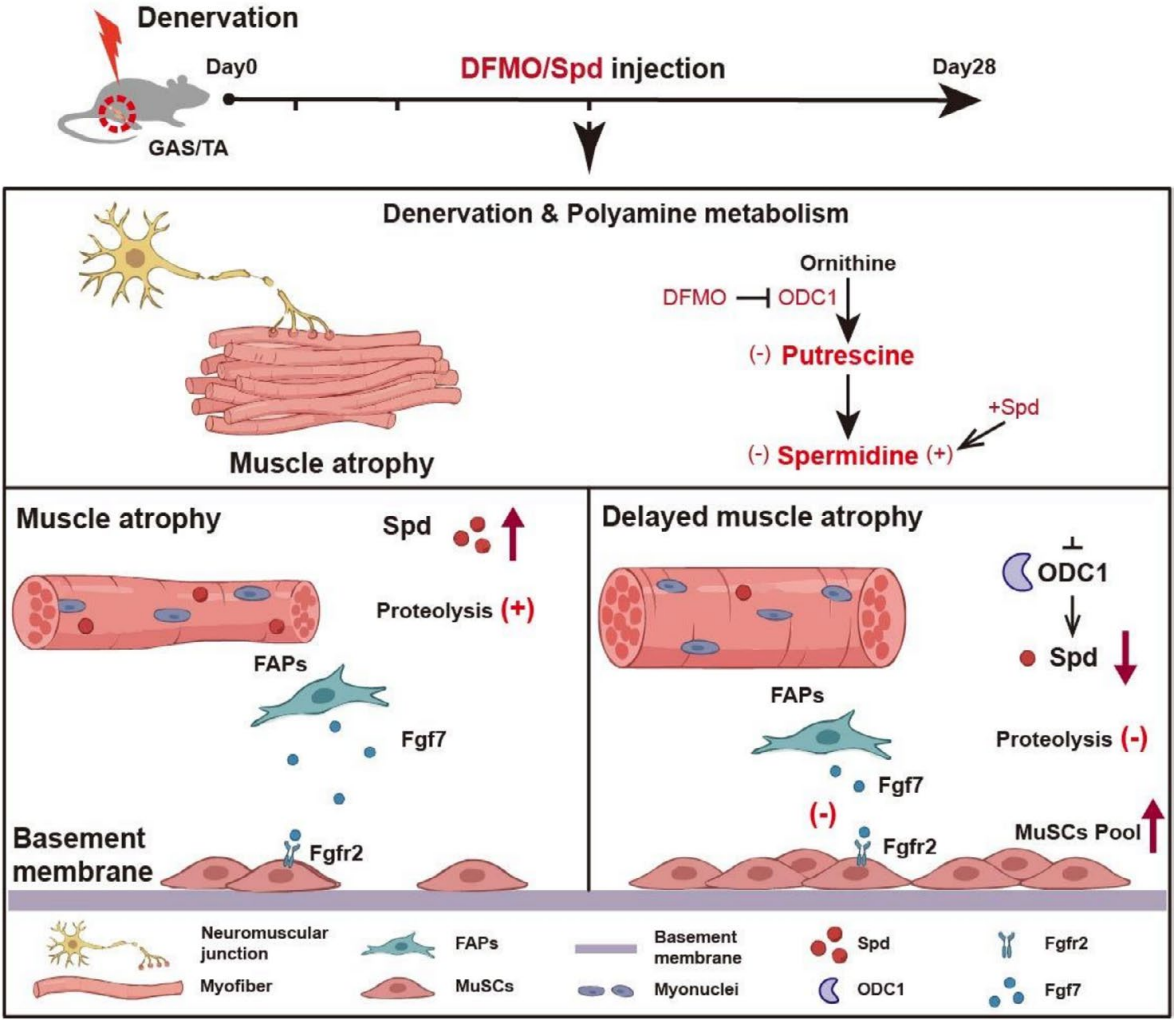
A schematic diagram illustrated the polyamine metabolism, and the effects of DFMO and spermidine in denervation‐induced muscle atrophy. −, iinhibition; +, promotion; DFMO, eflornithine; FAPs, fibro‐adipogenic progenitors; GAS, gastrocnemius; MuSCs, muscle stem cells; ODC1, ornithine decarboxylase; Spd, spermidine; TA, tibialis anterior.

Skeletal muscle is an important locomotor, endocrine and metabolic organ, which is susceptible to aging, malnutrition, cachexia, disuse and denervation, leading to muscle loss and weakness [34, 35]. Denervation‐induced muscle atrophy severely impairs muscle function, but lacks effective treatments. Our study identifies polyamine remodelling as a key metabolic feature of denervation‐induced skeletal muscle atrophy and provides mechanistic evidence that targeting polyamine biosynthesis alleviates muscle atrophy through coordinated effects on myofibers, MuSCs, and the stromal niche. By integrating phenotypic analysis, pharmacological intervention, single‐nucleus transcriptomics and cell–cell communication analysis, we propose that denervation‐induced activation of the polyamine pathway contributes to activation of the ubiquitin‐proteasome program and maladaptive niche signalling that promotes MuSCs overactivation and fibrogenic remodelling. Pharmacological ODC1 inhibition with DFMO reduces Spd levels, restrains proteolysis‐associated transcriptional responses, reprograms FAPs toward a less fibrogenic state, and attenuates FAP‐derived FGF7 signalling to MuSCs, thereby preserving the stem cell pool (Figure 7).

During denervation, the blockade of innervation regulates the gene expression of enzymes in the polyamine metabolism pathway, as well as the contents of polyamines [36, 37]. Researches showed that Spd and putrescine were elevated post denervation, especially increased Spd lasting for 8 weeks in response to denervation [38, 39]. Our study found that denervation induces a sustained increase in polyamines peaking around day 14, temporally aligning with the rapid phase of muscle mass loss. The strong induction of ODC1 and other biosynthetic enzymes, coupled with relatively modest changes in catabolic enzymes, supports the interpretation that denervation promotes net polyamine synthesis rather than simply altering turnover. Polyamines are intimately linked to translational capacity, chromatin accessibility, and stress responses [40]. Thus, their elevation could act as an upstream amplifier of transcriptional and proteostatic remodelling in denervated muscle. Importantly, our functional experiments refine this concept by implicating Spd as a pro‐atrophic signal, on account that Spd supplementation induced muscle atrophy and increased atrogenes expression in healthy muscle, and exacerbated denervation‐induced atrophy without markedly affecting fibrosis.

Spd, a kind of naturally occurring polyamine, widely existing in all organisms from plants to animals, is indispensable for growth and development [30]. However, Spd concentrations decrease with aging, usually causing the onset of age‐related diseases which are characterised as defect of autophagy and chronic inflammation [16, 41]. Spd participates in hypusination of eIF5A, regulates translation elongation, and can modulate autophagy [18, 42]. As a natural inducer of autophagy, the health‐promoting and anti‐aging effects of Spd, such as life span extension, tumour suppression, neuroprotection and cardiovascular protection, are linked to its ability to stimulate cytoprotective autophagy [30, 43]. In muscular diseases, researches showed that Spd reactivated autophagy to clear dysfunctional organelles in collagen VI‐null mice, ameliorating the histological and ultrastructural muscle defects [44, 45]. In addition, Spd and exercise attenuated D‐gal‐induced aging‐related skeletal muscle atrophy through enhancing autophagy in the AMPK‐FOXO3a signal pathway [46]. However, in denervated muscle atrophy, loss of innervation induces rapid muscle atrophy due to the greater protein degradation than protein synthesis, attributed to the activation of the ubiquitin‐proteasomal system and autophagy‐lysosomal system [47]. Researches showed that exogenous TGF‐β1 promoted denervated muscle atrophy by up‐regulating HMGB1 and increasing autophagy activity [48]. Meantime, researches showed that inhibition of autophagy by drug treatment prevented muscle atrophy; for example, catalpol, salidroside and celecoxib alleviated denervated muscle atrophy by inhibiting autophagy [49, 50, 51]. In this context, Spd further promoted the autophagic activity and aggravated muscle atrophy in healthy and denervated conditions, which suggested a detrimental role of Spd in denervation‐induced muscle atrophy. Consequently, the biological effects of Spd are highly context dependent. In addition, differences in dosage, timing and route of Spd exposure may also explain the divergent outcomes. Prior studies often used oral drinking water supplemented with Spd or intraperitoneal injection at relatively low doses [45, 46], whereas our study examined endogenous polyamine synthesis under denervation and used Spd to treat denervated muscle. It is therefore plausible that physiological or pharmacological replacement of Spd in a deficient state may be protective, whereas excessive or sustained Spd availability in denervated muscle may amplify autophagy to promote muscle atrophy. Based on these considerations, dose–response studies to define the threshold at which Spd transitions from protective to maladaptive and metabolic flux analysis to identify the dominant cellular sources deserves in‐depth investigation.

DFMO conferred robust protection against denervation‐induced pathology, improving muscle atrophy and fibrotic remodelling. Mechanistically, DFMO lowered intramuscular polyamines and restored Spd toward baseline. The associated decrease in atrogene expression supports that reduced Spd availability dampens proteolytic drive. While DFMO is classically viewed as an antiproliferative agent in cancer biology [52, 53], our data indicate that in denervated muscle DFMO does not impair regenerative capacity, as evidenced by increased MuSCs preservation. Our results may reflect context specificity of DFMO. Denervation triggers maladaptive overactivation of stress and niche signalling pathways, and suppression of polyamine‐driven activation may restore a more balanced homeostatic state. Consequently, DFMO is an attractive candidate because of its established pharmacology; however, optimal dosing, timing relative to denervation, and potential impacts will need careful evaluation.

Single‐nucleus RNA sequencing provided a high‐resolution view of the cellular states underpinning these phenotypes. Denervation resulted in depletion of type IIb myonuclei and expansion of an atrophic myonuclear state. These findings align with the concept that the type IIb myofiber is particularly vulnerable to denervation and that denervation simultaneously provokes degenerative and compensatory regenerative programs [54, 55]. DFMO reduced the proportion of atrophic myonuclei and increased MuSCs abundance. Given prior evidence that denervation can induce premature MuSCs activation and differentiation, this potentially led to depletion of the quiescent pool and impaired long‐term regenerative capacity [31, 56]. Increased MuSCs abundance in DFMO‐treated muscle could reflect reduced inappropriate activation and improved survival that preserves the reservoir. The upregulation of transcriptional programs related to cell number homeostasis is consistent with a shift toward maintaining balanced self‐renewal. Consequently, our results have important implications. Therapies that merely reduce myofiber proteolysis may slow atrophy, but preserving the MuSCs pool could improve recovery potential, particularly in clinical scenarios where reinnervation is delayed or incomplete.

Our niche‐focused analysis further indicates that the benefits of DFMO extend beyond myofiber‐intrinsic mechanisms. While denervation had relatively limited effects on immune cell proportions, DFMO reduced inflammatory transcriptional programs, including pathways related to leukocyte differentiation and cytokine responses. This suggests that DFMO may dampen inflammatory amplification that can exacerbate proteolysis and fibrosis. Notably, although the minimal fluctuations in immune cell proportions may reflect genuine tissue physiology, they must be interpreted cautiously due to the inherent limitations of snRNA‐seq in capturing rare immune populations within skeletal muscle. Consequently, future investigations utilising targeted enrichment methods, such as flow cytometry, are required to definitively isolate and profile these immune dynamics. In stromal compartments, FAPs emerged as the cell type most responsive to DFMO at the transcriptional level. DFMO downregulated gene programs linked to extracellular matrix assembly, fibroblast activation, and regulation of myoblast proliferation, aligning tightly with the observed reduction in collagen deposition and fibrotic remodelling. Our further cell communication analysis identified FGF signalling as a DFMO‐sensitive axis from FAPs to MuSCs and the dominant ligand‐receptor pair was FGF7‐FGFR2. Prior studies have shown that FAP‐derived FGF7 can promote satellite cell proliferation and differentiation [33], a response that can be beneficial after acute injury but may become maladaptive in denervation by driving premature activation and potential exhaustion of the stem cell pool. In our study, DFMO suppresses FAP‐derived FGF7‐FGFR2, thereby maintaining MuSCs homeostasis and supporting sustained regenerative capacity. Consequently, polyamine may influence FAP activation state and secretory programs, which in turn shape MuSCs behaviour. This ligand‐receptor pair can be a more targeted therapeutic strategy that could complement or substitute for systemic polyamine inhibition.

Together, our results provide a coherent framework linking metabolic remodelling to cell‐state transitions and niche communication in denervated muscle. Targeting these pathways may improve outcomes in denervation‐associated muscle atrophy and potentially other forms of neuromuscular degeneration.

## 5 Conclusion

Denervation triggers a sustained activation of polyamine biosynthesis in skeletal muscle, with Spd emerging as a key pro‐atrophic metabolite that enhances ubiquitin‐proteasome‐mediated proteolysis. Pharmacological inhibition of ODC1 by DFMO reduces Spd levels, reduces the ubiquitin‐proteosome system induction, delays muscle atrophy, and alleviates fibrotic remodelling. Single‐nucleus profiling further indicates that DFMO maintains the MuSCs pool and reprograms FAPs to suppress FGF7‐FGFR2 signalling to MuSCs. Thus, targeting polyamine metabolism represents a promising strategy against denervation‐induced muscle atrophy.

## Author Contributions

P.Y., P.T., M.Z. and F.C. contributed to the conception and design of the study. M.Z., F.C., S.G. and F.C. performed the statistical analysis. M.Z. and F.C. wrote the first draft of the manuscript. S.G., F.C., P.F., H.Z., M.C. and Y.L. wrote sections of the manuscript. All authors contributed to manuscript revision, read and approved the submitted version.

## Funding

This work was supported by National Science and Technology Major Project for the Prevention and Treatment of Cancer, Cardiovascular and Cerebrovascular Diseases, Respiratory Diseases, and Metabolic Diseases (2024ZD0530502).

## Ethics Statement

The animal study was approved by the Institutional Animal Care and Use Committee of Chinese PLA General Hospital (Approval number: 2021‐X17‐69). All experiments were performed in compliance with the national guidelines for the care and use of animals.

## Conflicts of Interest

The authors declare no conflicts of interest.

## Supporting information


**Figure S1:** DFMO improved denervation‐induced muscle atrophy. (A) Gross morphology of tibialis anterior harvested from different treatment groups at days 3, 7, 14 and 28 after denervation. Scale bar 5 mm. (B) The wet weight ratio of tibialis anterior at the indicated time points after denervation. (C) The normalised muscle weight of tibialis anterior at days 28 after denervation treated with saline or DFMO. (D) The mRNA expression levels of E3 ubiquitin ligases, *Trim63* and *Fbxo32*, by RT‐qPCR in gastrocnemius post denervation. (E) The quantification of fibrotic area of gastrocnemius at days 14 after denervation treated with saline or DFMO, related to Figure 2N. (F, G) The representative Masson‐Trichrome staining images and quantification of fibrotic area of gastrocnemius muscles at days 14 after denervation treated with saline or DFMO. Scale bar 50 μm. Dots are individual values of independent animals. Data were represented as mean ± SD. Statistical tests: two‐tailed unpaired Student's *t*‐test (B), one‐way ANOVA test (C, D, E, and G). * *p* < 0.05, ** *p* < 0.01, *** *p* < 0.001. *n* = 3–5/group.
**Figure S2:** Spd treatment aggravated denervation‐induced muscle atrophy. (A) Gross morphology of tibialis anterior harvested from different treatment groups at days 3, 7, 14 and 28 after denervation. Scale bar 5 mm. (B) The wet weight ratio of tibialis anterior at the indicated time points after denervation. (C) The myofiber size distribution of gastrocnemius at days 14 after denervation treated with saline or spermidine. (D, E) The expressions of E3 ubiquitin ligases MuRF‐1 and Atrogin‐1 quantified via western blotting in gastrocnemius at days 7 after denervation treated with saline or spermidine. (F, G) The representative Masson‐Trichrome staining images and quantification of fibrotic area of gastrocnemius at days 14 after denervation treated with saline or spermidine. Scale bar 100 μm. (H, I) The representative Sirius Red staining images and quantification of fibrotic area of gastrocnemius at days 14 after denervation treated with saline or spermidine. Scale bar 50 μm. Dots are individual values of independent animals. Data were represented as mean ± SD. Statistical tests: two‐tailed unpaired Student's *t*‐test (B), one‐way ANOVA test (C, E, G, and I). ns *p* > 0.05, * *p* < 0.05, *** *p* < 0.001. *n* = 3–5/group.
**Figure S3:** DFMO treatment inhibited proteolysis and autophagy in myofibers. (A) Differences in nuclei densities for Con, Den and DFMO‐treated denervated gastrocnemius. (B) Stacked bar plots depicting the relative proportion of each annotated population across the three groups. (C) Gene Ontology enrichment analysis of differentially expressed genes in MF comparing denervated and control muscles and DFMO‐treated vs. denervated muscles.
**Figure S4:** (A) The melting curves for qPCR, related to Figure 1M, Figure 3I,R, Figure 4K, and Figure S1D. (B) The raw and representative immunofluorescence images of gastrocnemius myofiber membrane, related to Figure 1F. (C) The raw and representative immunofluorescence images of spermidine in gastrocnemius, related to Figure 3C. (D) The raw and representative immunofluorescence images of MyHC‐stained myotubes, related to Figure 4K.

## Data Availability

The data that support the findings of this study are available from the corresponding author upon reasonable request.
