# Supplementary material for: Inhibition of Ornithine Decarboxylase 1 Mitigates Denervation‐Induced Muscle Atrophy by Suppressing Proteolysis and Preserving Muscle Stem Cell Homeostasis
Source: Cell Prolif. 2026 May 15;59(10):e70231. doi: 10.1111/cpr.70231 (PMC13325653; doi:10.1111/cpr.70231)
Supplement: Supplementary file 1 [file CPR-59-e70231-s001.docx]

**Figure S1**

DFMO improved denervation-induced muscle atrophy. (A) Gross morphology of tibialis anterior harvested from different treatment groups at days 3, 7, 14 and 28 after denervation. Scale bar 5 mm. (B) The wet weight ratio of tibialis anterior at the indicated time points after denervation. (C) The normalized muscle weight of tibialis anterior at days 28 after denervation treated with saline or DFMO. (D) The mRNA expression levels of E3 ubiquitin ligases, *Trim63* and *Fbxo32*, by RT-qPCR in gastrocnemius post denervation. (E) The quantification of fibrotic area of gastrocnemius at days 14 after denervation treated with saline or DFMO, related to Figure 2N. (F, G) The representative Masson-Trichrome staining images and quantification of fibrotic area of gastrocnemius muscles at days 14 after denervation treated with saline or DFMO. Scale bar 50 μm. Dots are individual values of independent animals. Data were represented as mean ± SD. Statistical tests: two-tailed unpaired Student’s t test (B), one-way ANOVA test (C, D, E, and G). * *P* < 0.05, ** *P* < 0.01, *** *P* < 0.001. n = 3-5/group.

**Figure S2**

Spd treatment aggravated denervation-induced muscle atrophy. (A) Gross morphology of tibialis anterior harvested from different treatment groups at days 3, 7, 14 and 28 after denervation. Scale bar 5 mm. (B) The wet weight ratio of tibialis anterior at the indicated time points after denervation. (C) The myofiber size distribution of gastrocnemius at days 14 after denervation treated with saline or spermidine. (D, E) The expressions of E3 ubiquitin ligases MuRF-1 and Atrogin-1 quantified via western blotting in gastrocnemius at days 7 after denervation treated with saline or spermidine. (F, G) The representative Masson-Trichrome staining images and quantification of fibrotic area of gastrocnemius at days 14 after denervation treated with saline or spermidine. Scale bar 100 μm. (H, I) The representative Sirius Red staining images and quantification of fibrotic area of gastrocnemius at days 14 after denervation treated with saline or spermidine. Scale bar 50 μm. Dots are individual values of independent animals. Data were represented as mean ± SD. Statistical tests: two-tailed unpaired Student’s t test (B), one-way ANOVA test (C, E, G, and I). ns *P* > 0.05, * *P* < 0.05, *** *P* < 0.001. n = 3-5/group.

**Figure S3**

DFMO treatment inhibited proteolysis and autophagy in myofibers. (A) Differences in nuclei densities for Con, Den and DFMO-treated denervated gastrocnemius. (B) Stacked bar plots depicting the relative proportion of each annotated population across the three groups. (C) Gene Ontology enrichment analysis of differentially expressed genes in MF comparing denervated and control muscles and DFMO-treated versus denervated muscles.

**Figure S4**

(A) The melting curves for qPCR, related to Figure 1M, Figure 3I, R, Figure 4K, and Figure S1D. (B) The raw and representative immunofluorescence images of gastrocnemius myofiber membrane, related to Figure 1F. (C) The raw and representative immunofluorescence images of spermidine in gastrocnemius, related to Figure 3C. (D) The raw and representative immunofluorescence images of MyHC-stained myotubes, related to Figure 4K.
